# Do the risk factors for type 2 diabetes mellitus vary by location? A spatial analysis of health insurance claims in Northeastern Germany using kernel density estimation and geographically weighted regression

**DOI:** 10.1186/s12942-016-0068-2

**Published:** 2016-11-03

**Authors:** Boris Kauhl, Jürgen Schweikart, Thomas Krafft, Andrea Keste, Marita Moskwyn

**Affiliations:** 1 Department of Medical Care, AOK Nordost – Die Gesundheitskasse, Berlin, Germany; 2 Department III, Civil Engineering and Geoinformatics, Beuth University of Applied Sciences, Berlin, Germany; 3 Department of Health, Ethics and Society, School of Public Health and Primary Care (CAPHRI), Faculty of Health, Medicine and Life Sciences, Maastricht University, Maastricht, The Netherlands

**Keywords:** Type 2 diabetes mellitus, Healthcare, Germany, Spatial analysis, Geographically weighted regression, Kernel Density Estimation, SaTScan, Street-level, big data

## Abstract

**Background:**

The provision of general practitioners (GPs) in Germany still relies mainly on the ratio of inhabitants to GPs at relatively large scales and barely accounts for an increased prevalence of chronic diseases among the elderly and socially underprivileged populations. Type 2 Diabetes Mellitus (T2DM) is one of the major cost-intensive diseases with high rates of potentially preventable complications. Provision of healthcare and access to preventive measures is necessary to reduce the burden of T2DM. However, current studies on the spatial variation of T2DM in Germany are mostly based on survey data, which do not only underestimate the true prevalence of T2DM, but are also only available on large spatial scales. The aim of this study is therefore to analyse the spatial distribution of T2DM at fine geographic scales and to assess location-specific risk factors based on data of the AOK health insurance.

**Methods:**

To display the spatial heterogeneity of T2DM, a bivariate, adaptive kernel density estimation (KDE) was applied. The spatial scan statistic (SaTScan) was used to detect areas of high risk. Global and local spatial regression models were then constructed to analyze socio-demographic risk factors of T2DM.

**Results:**

T2DM is especially concentrated in rural areas surrounding Berlin. The risk factors for T2DM consist of proportions of 65–79 year olds, 80 + year olds, unemployment rate among the 55–65 year olds, proportion of employees covered by mandatory social security insurance, mean income tax, and proportion of non-married couples. However, the strength of the association between T2DM and the examined socio-demographic variables displayed strong regional variations.

**Conclusion:**

The prevalence of T2DM varies at the very local level. Analyzing point data on T2DM of northeastern Germany’s largest health insurance provider thus allows very detailed, location-specific knowledge about increased medical needs. Risk factors associated with T2DM depend largely on the place of residence of the respective person. Future allocation of GPs and current prevention strategies should therefore reflect the location-specific higher healthcare demand among the elderly and socially underprivileged populations.

## Background

The prevalence of chronic diseases and therefore the projectable utilization of healthcare depend strongly on the demographic and socio-economic composition of the respective population [1–3]. International studies suggest a strong relationship between the proportion of elderly, low socio-economic status and a higher prevalence of chronic diseases [2, 4–6]. However, planning of GPs in Germany still relies mainly on the ratio of inhabitants to GPs at fairly large scales [7] and does neither sufficiently reflect the location-specific higher prevalence of chronic diseases among the elderly and population groups with a lower socio-economic status, nor the accessibility of GPs in rural areas [8].

With the ongoing demographic transition and migration processes from rural to urban areas, the gap between demand and supply of health care is already widening in Germany. While the ageing of the population and therefore the prevalence of chronic diseases increases in rural areas, the availability of GPs decreases [9]. To meet the increased demand for healthcare especially in rural areas, it is important to identify locations with higher healthcare demand as spatially precise as possible. Additional knowledge about the population groups, which are most at risk in specific locations is necessary to effectively plan the future provision of GPs and immediate preventive measures where they are needed most.

Type 2 Diabetes Mellitus (T2DM) is a major public health threat with an increasing prevalence among the general population worldwide [4, 10, 11] and especially in Germany [3]. Prevention and access to healthcare are necessary not only to prevent a further increase but also to prevent severe complications such as lower-extremity amputation [12] or stroke [10].

Despite behavioral risk factors such as lack of physical exercise, dietary deficits and smoking [13], a wide range of studies additionally highlights an association between age, lower socioeconomic status and T2DM [4, 14–16].

Geographic information systems (GIS) and spatial regression models at the ecological level have gained increasing attention in recent years as this approach allows an analysis of possible risk factors that are often unavailable on an individual level due to privacy restriction [15, 17]. For T2DM, this approach might help to identify the population groups, which are most in need for the provision of healthcare and access to preventive measures. However, several studies point out that socio-demographic risk factors for T2DM, but also for a wide range of other diseases depend largely on the place of residence of the respective individual [4, 14, 15, 17, 18]. As a consequence, a one-size fits all solution seems therefore inappropriate for effective public health strategies and allocation of healthcare [15].

Analyzing the spatial distribution of T2DM and associated risk factors in Germany is challenging, as epidemiological data on chronic diseases is seldom publicly available [19]. Only few studies have examined the spatial distribution of T2DM in Germany [16, 20–23]. However, the majority of these studies are based upon data from Germany’s largest telephone survey of the Robert-Koch-Institute (GEDA) [16, 20, 21]. A spatial analysis of this data source is therefore restricted to fairly large areas such as the counties in Germany [16, 21], or includes only a selection of municipalities [20]. Analyses based on surveys however, tend to underestimate the prevalence of T2DM as persons with a higher socioeconomic status are more likely to respond than persons with a lower socioeconomic status [20, 21]. Therefore, such surveys have only limited use for a demand-driven planning and allocation of healthcare and prevention strategies.

Health insurance in Germany is generally mandatory and approximately 86% of the population are covered by one of the statutory health insurance providers, 10% are covered by private health insurance providers and the remaining 4% are covered by the state [24]. However, there are large socio-demographic differences between members of the various statutory health insurances [25]. As the provision and allocation of primary healthcare in Germany is planned and organized by the association of statutory health insurance physicians in accordance with the statutory health insurance providers [7], it is necessary for each health insurance provider to engage in planning of primary healthcare based on an empirical evaluation of the medical demand of their respective insurants.

At the federal level, 1671 inhabitants per 1 GP at the spatial scale of central areas (Mittelbereiche) of the Federal Agency of Building and Urban Development (BBSR) is the target-ratio for the allocation of GPs in Germany [7]. The association of statutory health insurance physicians defines over- or undersupply as deviation from this ratio by 110 and 50%, respectively and has to undertake appropriate measures if over- or undersupply exists [7]. However, this ratio was established in the 1990s [7] and does not recognize an increased prevalence of T2DM and other chronic diseases in location-specific population groups. The association of statutory health insurance physicians has reacted to this criticism by incorporating a demographic factor and allowing deviations from the established inhabitants to GP ratio for areas with increased medical demand in their revised planning guidelines [7]. However, due to the lack of reliable, small-scale public health data on chronic diseases, an increased medical demand of a location-specific population group is still difficult to detect [16, 20–23]. To realistically capture such an increased demand for healthcare, more reliable sources than survey data and spatial analyses at smaller scales are necessary than it is currently possible with survey data in Germany.

In this context, health insurance claims of the AOK Nordost have several advantages over survey data: (a) This data source represents a large sample of northeastern Germany’s population, (b) can be analyzed on a fine geographic scale and (c) prevalence estimates of health insurance claims are not depending on the response rate of participants and are therefore a more realistic estimate of the “true” prevalence of chronic conditions than survey data [26]. Ultimately, a spatial analysis of this data source might provide new and inclusive insights on the spatial distribution of chronic diseases and population-based risk factors.

The goal of our paper is therefore to (1) analyze the spatial distribution of T2DM based on health insurance claims of northeastern Germany’s largest statutory health insurance provider; (2) to evaluate possible risk factors using global ecological regression models and (3) to examine the spatially varying association between socio-demographic risk factors and T2DM.

## Methods

### Dependent variable

In this study, we used data from northeastern Germany’s largest statutory health insurance provider (AOK Nordost) for 2012, which covers roughly 1.79 million persons (approximately one quarter of the population) of which 361 thousand persons are diagnosed with Type 2 Diabetes.

Persons diagnosed with T2DM were defined in our study as having a confirmed diagnosis of T2DM (ICD-10: E11.-). As long as the insurant is treated for T2DM, this diagnosis will remain in the insurant’s personal medical file as the diagnosis is renewed with each GP visit associated with T2DM. To ensure that each insurant and diabetic is included only once in the analysis, the unique insurant number was used to exclude possible double entries within the database from the analysis.

The data was anonymized and was geocoded based on exact street-level data using the ESRI ArcGIS geocoder. The data included only age in broad age categories (0–5, 6–11, 12–17, 18–24, 25–44, 45–64, 65–79 and 80 and older) and the address coordinates. We used a step-wise geocoding process where the data was first geocoded based on the exact street address where possible (90.2%). Of the remaining data, 6.7% were matched to the centroids of the street and 3.1% were matched to the postal code centroids. The address coordinates for Berlin were obtained from the Senatsverwaltung für Stadtverwaltung Berlin; the address coordinates for Brandenburg were obtained from the Landesvermessungsamt und Geobasisinformation Brandenburg (Geobasisdaten © GeoBasis-DE/LGB 2016, GB-D 13/16) and the coordinates for Mecklenburg-Vorpommern were obtained from the Landesamt für Innere Verwaltung, Amt für Geoinformation, Vermessungs- und Katasterwesen (Geobasisdaten © GeoBasis-DE/M-V 2016).

### Explanatory variables

In this study, we assessed a wide range of demographic, socioeconomic and variables related to the physical environment for their association with T2DM. Demographic variables were calculated based on the proportion of AOK insurants per demographic group. Socioeconomic variables included the proportion of unemployed persons in different age groups, information on taxation, land use, household composition and a wide range of other indicators. Variables related to the physical environment included the proportion of green spaces, recreational spaces and built surfaces. The data were obtained for the year 2012 from the INKAR database of the Federal Agency of Building and Urban Development (BBSR). Data on marital status, household and family composition were obtained from the census 2011 for Germany. All data were available on the spatial scale of the association of municipalities. Additionally, we included data on the spatial distribution of GPs in our analysis to examine whether the availability of healthcare influences the prevalence of T2DM. We included two variables: The proportion of inhabitants to GPs and the average distance to GPs. The average distance to GPs was calculated based on the driving distance of each insurant to the closest GP and was then aggregated to match the association of municipalities. The street network dataset was downloaded from OpenStreetMap [27]. The association of municipalities in Germany was chosen as the unit of analysis as this is the smallest spatial scale, for which a wide range of indicators is available without areas being omitted due to privacy protection as it would be the case for municipalities. However, this scale does not allow an analysis of intra-urban differences as the indicators of BBSR are not available for a smaller administrative unit than the association of municipalities.

### Statistical analysis

#### Bivariate kernel density estimation

In this study, we used a bivariate, adaptive kernel density estimation (KDE) to display the spatial heterogeneity of T2DM independent of administrative boundaries. In most epidemiological studies, disease and population data are only available for aggregated data such as postal codes, municipalities, counties or districts [10, 16, 21, 28]. However, problems arise in the detection of local clusters and associations to socio-demographic exposure factors due to the relatively arbitrary shape and quantity of spatial units, which is often referred to as the “modifiable area unit problem” [29]. This may be especially misleading in rural areas where administrative boundaries are very large. As a consequence, a cartographic visualization of disease risk without the restrictions of artificially created boundaries is favorable.

Bivariate kernel density estimation has been previously applied in small-scale studies for HIV [30, 31], cancer [32, 33], Alzheimer [34] and crime intensity [35] and thus seems useful for a small-scale analysis of T2DM as well.

A major concern when applying a bivariate KDE is the choice of bandwidth. If the bandwidth is too small, rates become highly unstable and spatial patterns are difficult to detect. If the bandwidth is too large, the map appears to be over smoothed and local extremes are smoothed away [33]. Although several statistical models exist to calculate the “optimal” bandwidth, such as the Likelihood Cross Validation [33, 36, 37], Least Squares Cross Validation [33, 38], Biased Cross Validation [33, 39], Smoothed Cross Validation [33, 40], or the direct plug-in method [33, 41], these aforementioned bandwidth selection models are generally only available for fixed bandwidth types [33].

As our study area comprises highly densely populated urban areas such as Berlin, Potsdam or Schwerin while at the same time comprising a large proportion of very sparsely populated rural areas, a KDE employing a fixed bandwidth would deliver no stable results. We therefore favored an adaptive bandwidth, which accounts for the varying population densities within our study area [32, 33].

Although a wide range of selection methods exist for a fixed bandwidth, automated procedures to select an optimal number of points to be included in an adaptive bandwidth for a bivariate KDE are scarce and are not yet fully satisfactory [33]. As there are no definite recommendations to define a bandwidth for a bivariate KDE, we therefore visually evaluated several possible combinations of minimum sample points [42, 43]. Including at least 0.1% of T2DM cases and 0.1% of insurants delivered the most useful results. The T2DM prevalence was therefore calculated as the ratio of at least 361 T2DM cases per km^2^ to 1791 insurants per km^2^. Given the varying population densities, the kernel was thus smaller in highly populated areas and larger in sparsely populated rural areas. In this study, we used a Gaussian kernel as it tends to produce more robust results than a kernel type with a definite boundary [43].

The calculation of the bivariate KDE was carried out using the CrimeStat IV software [43]. The results were then imported in ESRI ArcGIS 10.3.

#### Sex- and age-standardization of prevalence rates

The bivariate, adaptive kernel density estimation allows a visualization of T2DM prevalence without the limitations of administrative areas but has the disadvantage of not being able to incorporate sex- and age-standardization.

To further facilitate interpretation of the spatial variations in T2DM prevalence, we directly adjusted for sex and age using the WHO standard population from 1976 [44] based on the five-digits postal codes of our study area. As the number of insurants between the five-digits postal code varies considerably, we applied spatial empirical Bayesian smoothing to borrow strength from neighboring postal codes to estimate more stable prevalence rates [45]. Neighboring areas were defined as postal codes sharing a common edge or boundary [46]. The computation was carried out in GeoDa 1.2.0 and the results were then imported in ESRI ArcGIS 10.3.

#### Cluster detection

The aim of cluster detection in our study was to evaluate whether a statistically significant elevated risk exists in certain areas. A purely visual inspection of the KDE and the adjusted rates would be misleading, as it is not possible to examine the number of cases behind the estimated rates alone. Applying a local cluster test on health data is important to prioritize areas for future public health interventions [30, 47] and has been previously shown useful to locate new clinics for chronically ill patients for diabetic kidney patients [48].

In this study, we used the spatial scan statistic (SaTScan). The spatial scan statistic is a local cluster test, which determines the location and significance of local clusters. This is achieved by a circular scanning window, which moves over the coordinates of the study area and evaluates all possible cluster locations and cluster sizes up to either a user defined maximum or the default settings of including up to 50% of the population at risk inside a cluster [30, 49]. The statistical significance is calculated using 999 Monte-Carlo replications [50]. We applied a purely spatial Poisson model, where the T2DM cases per coordinate/sex- and age-adjusted number of T2DM cases per postal code were assigned as cases and all insurants per coordinate/postal code were assigned as population [30, 49, 50]. The maximum cluster size was restricted to a maximum radius of 10 km. This was done as (a) the standard setting of including up to 50% of the population at risk often produces results of no practical use [51] and (b), we defined 10 km as the maximum reasonable driving distance to GPs in rural areas of northeastern Germany. For the analysis of the point data, we used the exact street-level coordinates and for the cluster analysis of the sex- and age-adjusted rates we used the centroid coordinates of the postal codes. The analysis was carried out using SaTScan v9.4.2.

### Spatial regression modelling

#### Ordinary least squares regression modelling

To create a meaningful and correct specified geographically weighted regression model (GWR), we first aimed to identify all possible explanatory variables through the global ordinary least squares (OLS) regression model. To achieve this, we first performed a natural log-transformation of the T2DM prevalence to satisfy the assumption of the OLS model that the dependent variable has to be normally distributed [52]. We used the raw rate instead of the age-adjusted T2DM prevalence as we specifically wanted to model the effect of older age groups on the T2DM prevalence.

We then compared the association between each potential explanatory variable and T2DM prevalence through univariate OLS regression models. As a large number of explanatory variables were found to be significantly associated to T2DM, we used a data-mining tool called “exploratory regression” in ESRI ArcGIS 10.3 to determine all possible variable combinations. This tool is comparable to a step-wise regression. It evaluates all possible variable combinations based on four criteria: (1): the coefficients are statistically significant; (2): the explanatory variables are free from multicollinearity; (3): the residuals are normally distributed and (4): the residuals are not spatially autocorrelated [52–54].

We then determined overall model significance, autocorrelation of the residuals, the presence of heteroscedasticity and a wide range of other diagnostics by creating an OLS model in ESRI ArcGIS 10.3. with the same explanatory variables as suggested by the exploratory regression that were found to deliver a plausible explanation of the T2DM prevalence.

#### Geographically weighted regression modelling

The OLS model is a global model, it therefore estimates only one single coefficient per explanatory variable averaged over the entire study area. However, the socio-demographic composition of the population in northeastern Germany varies strongly at the local level. It is therefore unlikely that the association between socio-demographic explanatory variables and T2DM is realistically reflected by a global regression model. Previous studies applying GWR on Diabetes [4, 15] as well as on a wide range of other diseases [18, 55, 56] pointed out that the correlations between explanatory variables and T2DM vary strongly across space. We therefore hypothesize that this applies to our study area as well. The GWR methodology is an extension to the standard regression models and estimates a wide range of local parameters to reflect changes over space in the association between an epidemiological outcome and explanatory variables [57].

Similar to the OLS model, we used the log-transformed T2DM prevalence as the dependent variable with the same explanatory variables that were found to be significant in the OLS model.

We used the centroids of the association of municipalities as the input coordinates. Similarly to the KDE, the GWR methodology uses a circular kernel to calculate the local estimates. The GWR model fits for each coordinate a regression equation where the coordinates in the center of the kernel are the regression points. The data points inside the kernel are then weighted with decreasing weights from the center towards the edge of the kernel. The bandwidth of the kernel can be either fixed or adaptive and the shape of the kernel can follow a Gaussian or a bi-square distribution. The optimization of the bandwidth can be based on one of the four available criteria: (1) Akaikes Information Criterion (AIC); (2) Akaikes corrected Information Criterion (AICc); (3) Bayesian Information Criterion (BIC) and (4) Cross Validation (CV) [57, 58]. We thus evaluated all 14 possible combinations of kernel shape, bandwidth type and bandwidth optimization method. The models without clustered residuals were further considered and out of those, the model with the lowest AICc value and highest adjusted R^2^ was then chosen as the final model. The calculation of the GWR model was carried out in the GWR4 software. To enhance visualization of the spatially varying coefficients, we used the software’s “prediction at non-sample points” function and calculated the predicted values for a grid of northeastern Germany based on a cell size of 100 m × 100 m. The obtained values were then interpolated using ordinary kriging in ESRI ArcGIS 10.3.

### Ethics statement

The data and results used in this study were anonymized and do not contain any personal information. The use of anonymized data for research purposes does not require a vote by an ethics committee or an institutional research board.

## Results

### Spatial distribution of T2DM

**Fig. 1:**
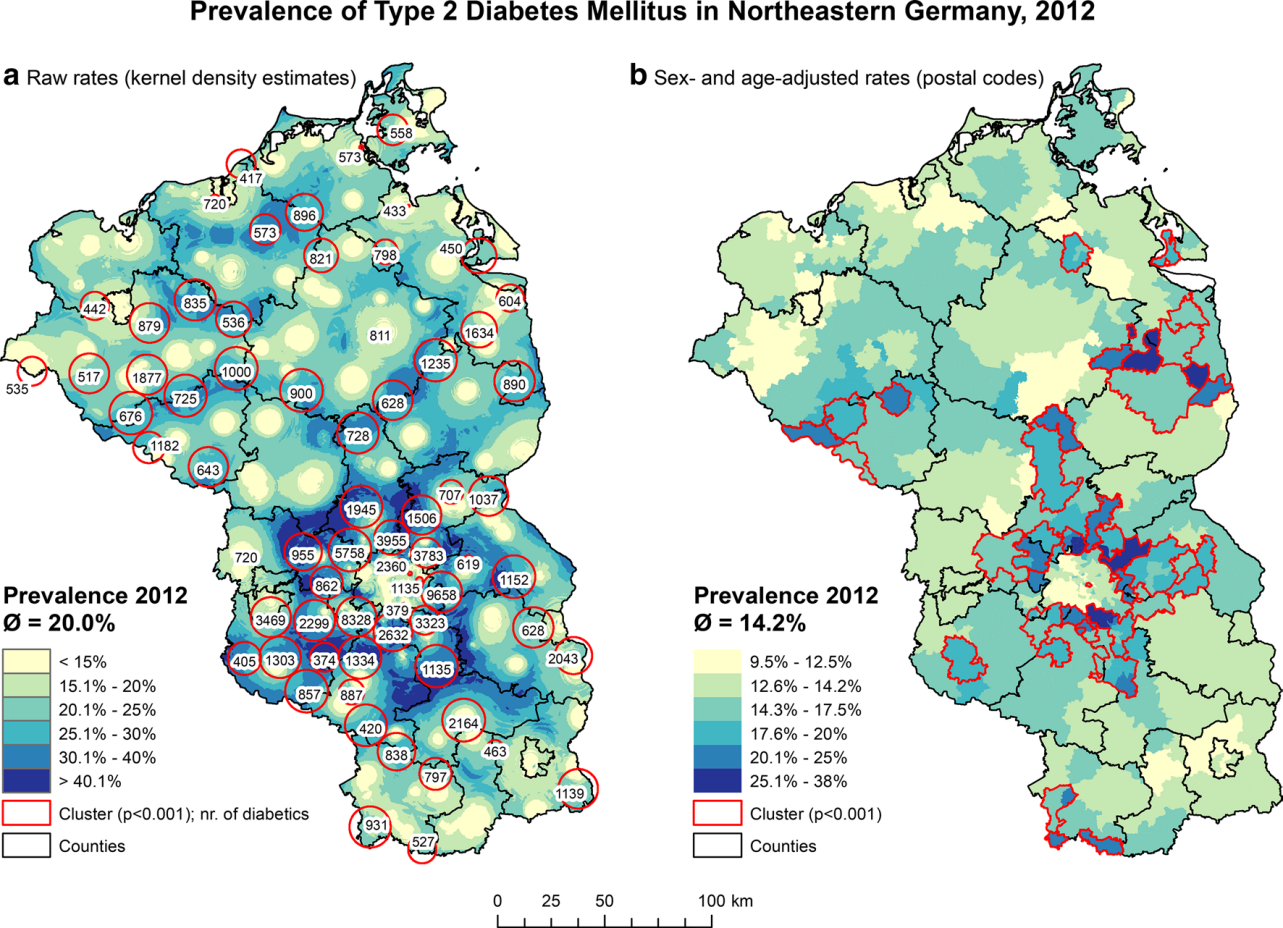
The spatial distribution of T2DM in northeastern Germany represented as **a** KDE estimates of the raw rate and **b** sex- and age-adjusted rates based on the five-digit postal codes

The overall raw prevalence of T2DM was 20.0% and the sex- and age-adjusted prevalence was 14.2%. However, the prevalence varied widely within the study area (Fig. 1). Generally, the prevalence was relatively low in the center of larger villages or urban areas and increased towards remote, rural areas. The highest prevalence and clusters with most cases could be observed in a ring in Brandenburg, surrounding Berlin. In Mecklenburg-Vorpommern, the number of clusters as well as the number of cases inside local clusters was lower than in Brandenburg.

### Socio-demographic risk factors of T2DM

Six variables were identified as significant predictors for T2DM in northeastern Germany (Table 1): (1) proportion of persons aged 65–79, (2) proportion of persons aged 80 and older, (3) proportion of unemployed persons aged 55–65; (4) proportion of employed persons which are subject to social insurance contribution, (5) mean income tax and (6) proportion of non-married couples, which live together in the same household. These six variables explained 44% of the variation in T2DM prevalence (Table 1). However, the residuals were clustered, reflecting that a global OLS model is not suitable to model the prevalence of T2DM.

**Table 1:** Results of the global OLS regression model

Variable	Coefficient	VIF
Intercept	2.259540***	
Persons aged 65–79 (%)	0.027251***	1.656689
Persons aged 80 and older (%)	0.010704**	1.650654
Unemployed persons aged 55–65 (%)	0.013354***	2.593295
Employed persons (%)	−0.006181**	1.602619
Mean income tax	0.000780**	2.272369
Non-married couples (%)	0.014524*	1.452730
Adjusted R^2^	0.44	
AICc	−313	
Global Moran’s I of residuals	I = 0.264 (p < 0.001)	

Significance levels: * ≤ 0.05; ** ≤ 0.01; *** ≤ 0.001

### Spatially-varying risk factors of T2DM

By comparing all 14 possible combinations of bandwidth type, kernel shape and optimization methods in terms of their AICc value, adjusted R^2^ and Moran’s I of the residuals (Table 2), the model using an adaptive bandwidth with a bi-square kernel shape and an AIC optimized bandwidth selection method fulfils the requirements of the residuals not being clustered and has the best model fit, both, in terms of the AICc value and adjusted R^2^. This model explains 66% of the spatial variations of T2DM prevalence and has a much better fit (AICc: −374) than the global OLS model (AICc: −313). This suggests that a local model is more suitable to model the socio-demographic risk factors for T2DM than a global model.

**Table 2:** Comparison of bandwidth types, kernel shapes and bandwidth optimization methods

Modell (bandwidth type, kernel shape, optimization method)	AICc	Adjusted R^2^	Moran’s I of residuals
Adaptive, Gaussian, AICc	−347	0.51	p < 0.001
Adaptive, Gaussian, AIC	−347	0.51	p < 0.001
Adaptive, Gaussian, BIC	−315	0.44	p < 0.001
Adaptive, Gaussian, CV	−347	0.51	p < 0.001
Fixed, Gaussian, AICc	−385	0.62	p < 0.05
Fixed, Gaussian, AIC	−265	0.66	p > 0.05
Fixed, Gaussian, BIC	−316	0.44	p < 0.001
Fixed, Gaussian, CV	−370	0.64	p > 0.05
Adaptive, bi-square, AICc	−394	0.63	p < 0.001
Adaptive, bi-square, AIC	−374	0.66	p > 0.05
Adaptive, bi-square, BIC	−320	0.45	p < 0.001
Fixed, bi-square, AICc	−385	0.62	p < 0.01
Fixed, bi-square, AIC	40	0.68	p > 0.05
Fixed, bi-square, BIC	−316	0.44	p < 0.001

**Fig. 2:**
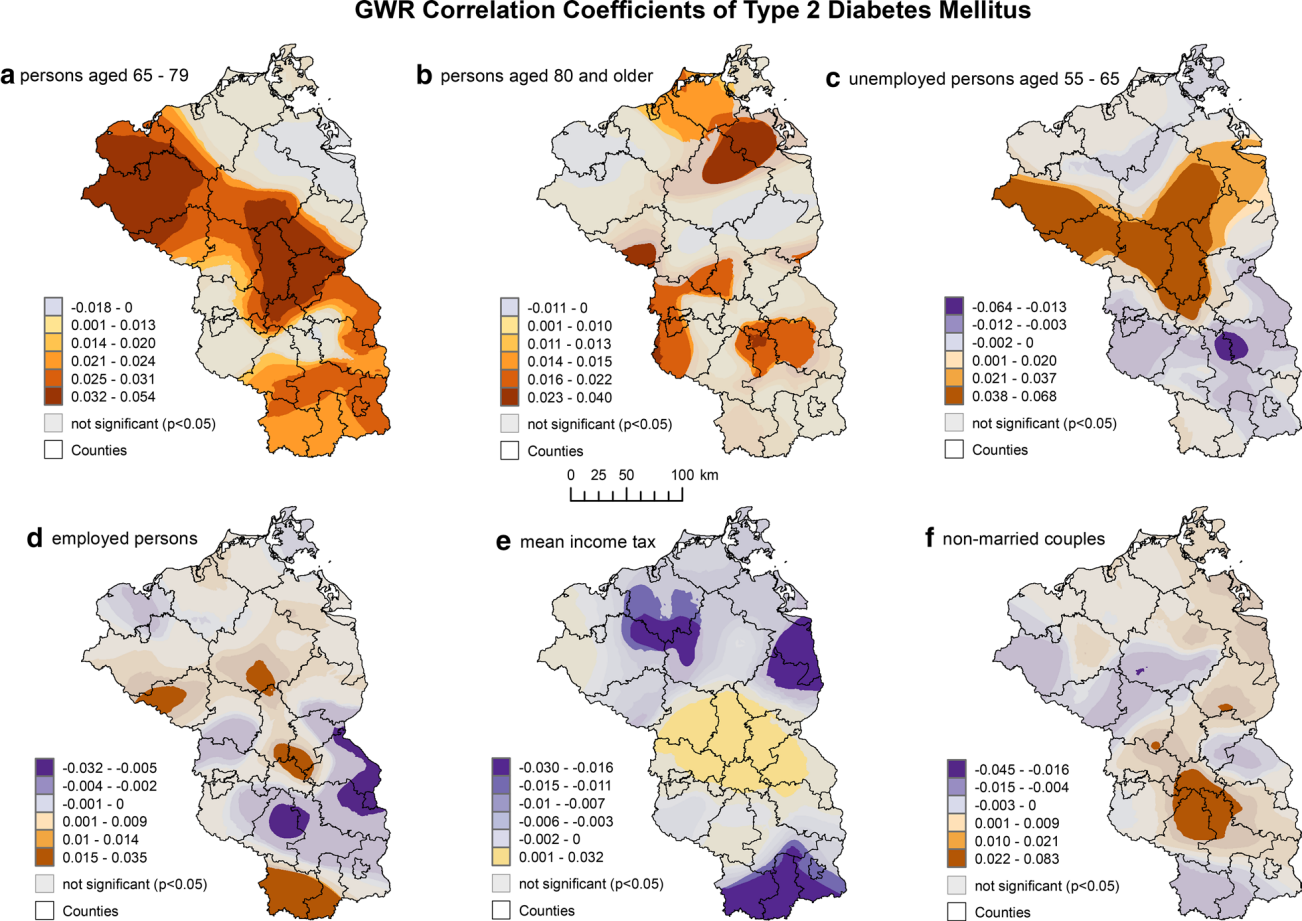
GWR correlation coefficients of type 2 diabetes mellitus for **a** persons aged 65–79, **b** persons aged 80 and older, **c** unemployed persons aged 55–65, **d** employed persons, **e** mean income tax and **f** non-married couples

The cartographic visualization of the GWR regression coefficients revealed strong regional differences of the association between the examined socio-demographic variables and T2DM prevalence (Fig. 2).

The impact of proportion of persons aged 65–79 was strongest in the areas north of Berlin in Brandenburg and two districts in the western part of Mecklenburg-Vorpommern. In these areas, 1% increase in persons aged 65–79 will increase the prevalence of T2DM between 3.2 and 5.4%. The association between persons aged 65–79 and T2DM prevalence was not significant in several districts west of Berlin and the northeastern districts in Mecklenburg-Vorpommern.

The association to proportion of persons aged 80 and older was significant in those areas where persons aged 65–79 were not significant with the exception of the islands Rügen and Usedom. The strongest impact could be observed in parts of the districts Vorpommern-Greifswald, Mecklenburgische Seenplatte and Prignitz. In these areas, 1% increase in persons aged 80 and older will increase the T2DM prevalence between 2.3 and 4%.

Unemployment rate among persons aged 55–65 was a significant positive predictor in several districts north of Berlin in Brandenburg and Mecklenburg-Vorpommern. In these areas, 1% increase in unemployment among the 55–65 year olds will increase the prevalence of T2DM between 3.8 and 6.6%. A significant negative association could only be observed in a small part of the districts Oder-Spree and Dahme-Spreewald. 1% decrease of unemployment among the 55–65 year olds will increase the T2DM prevalence between 1.3 and 6.4%.

The association between proportion of employed persons, which are subject to social insurance contribution, and T2DM changed sign across the study area. In the areas, where the proportion of employed persons was significant positively associated, 1% increase in employed persons was associated with 1.5–3.5% increase in T2DM prevalence. In the areas where the proportion of employed persons was significant negatively associated, 1% decrease of employed persons was associated with a 0.5–3.2% increase in T2DM prevalence. However, the association between employed persons and T2DM was only significant in a fraction of areas.

Similar to proportion of employed persons, the association between mean income tax and T2DM changed sign across the study area. In several districts north of Berlin, where the association between income tax and T2DM prevalence was positive, 10 Euro income tax per person per year will increase the T2DM prevalence by 0.1–3.2%. In the areas where the association to income tax was significant negative, 10 Euro less income tax per person per year will increase the T2DM prevalence between 1.6 and 3%.

The proportion of non-married couples sharing a common flat was only significant in several small parts of the districts Dahme-Spreewald and Teltow-Fläming. In these areas, 1% increase in non-married couples will increase the T2DM prevalence between 2.2 and 6.3%.

## Discussion

The prevalence of T2DM varies strongly at the very local level and clusters especially in rural areas in Brandenburg and Mecklenburg-Vorpommern. Socio-demographic risk factors consisted of proportion of persons aged 65–79, proportion of persons aged 80 and older, unemployment rate among the 55–65 year olds, proportion of employed persons, which are subject to social insurance contribution, mean income tax and proportion of non-married couples sharing a common flat. However, all associations displayed strong regional differences.

The overall prevalence of T2DM was 20%. After adjusting for sex and age, the prevalence of 14.2% was still higher than national estimates based on data derived from the telephone survey of the Robert-Koch-Institute (GEDA), which estimated the prevalence of known Diabetes to be at 8.8% among adults in Germany [3]. However, estimates derived from surveys such as the GEDA study are rather underestimated as healthy participants are more likely to respond than chronically ill patients [20, 21]. In this study, the estimated prevalence exceeds these previous estimates by far. As our study area comprises the most deprived areas in Germany [28], it is not surprising that our estimates exceed those of the GEDA study. Additionally, the proportion of older inhabitants, persons with low levels of education and unemployed persons among the local AOK health insurances is generally higher than in other statutory health insurances. As a logical consequence, the prevalence of chronic diseases is higher in our population sample than in the rest of the population [25].

The spatial distribution of T2DM varied strongly and formed clusters on small geographic scales. This was reflected by the results of the bivariate kernel density estimation and the results of the spatial scan statistic. Spatial heterogeneity and local clustering is typical for a wide range of chronic diseases [12, 59–62]. Our results are therefore in line with other studies but add an important level of spatial detail to previous research. The combination of the bivariate KDE and the spatial scan statistic complimented each other fairly well using the settings chosen in this study. However, we had to use a very conservative *p* value for the cluster analysis, as the number of clusters using a p-value of 0.05 was simply too high to allow a detailed investigation.

We identified six risk factors for T2DM in northeastern Germany: (1) proportion of persons aged 65–79, (2) proportion of persons aged 80 and older, (3) proportion of unemployed persons aged 55–65; (4) proportion of employed persons which are subject to social insurance contribution, (5) income tax and (6) proportion of non-married couples, which live together in the same household.

The association of T2DM to older age groups was expected as T2DM displays a strong association to older age groups [3, 4, 22]. The association of T2DM to the proportion of persons aged 65–79 and persons aged 80 and older is therefore in line with these studies although these associations were not in the entire study area significant.

Several studies pointed out that T2DM is associated with a lower socio-economic status [4, 14–16]. This is reflected by the strong association of unemployed persons aged 55–65 to T2DM. Given the high proportion of older persons among the AOK insurants, it is not surprising that specifically the unemployment rate among persons aged 55–65 was significant, but not unemployment rate in general. Additionally, this reflects the value of stratified socio-economic data as these findings could allow a more targeted prevention strategy among the at-risk population group.

The association to employed persons, which are subject to social insurance contribution, has to be seen in the context of income tax. Employed persons were positively associated in the areas, where income tax was negatively (but not significant) associated with T2DM prevalence. This reflects the association of T2DM to the lower-income groups [4, 15] and thus highlights the importance of determining location-specific association for T2DM. The negative association of employed persons to T2DM in specific areas can in part be explained by the exclusion criteria of employed persons in Germany. Excluded under this definition are for example persons working in marginal employment, soldiers, self-employed persons, non-working family members and government officials [63]. Given the association of T2DM to lower socio-economic status, these results might indicate that in areas where the association to employed persons is negative, persons working in marginally employment and non-working family members are at major risk for T2DM.

Although income tax was overall positively associated to T2DM, the results of GWR point out that income tax was in several areas significant negatively associated, confirming the results of previous studies [4, 15]. The positive association of income tax to T2DM prevalence is very specific to the area surrounding Berlin, which is often referred to as the commuter belt. This positive association reflects that in specific areas, a higher income may pose a risk factor for T2DM as well.

Several studies have shown that marital status has an effect on the overall health of the population. An unmarried status is often associated with a higher prevalence of chronic diseases and premature death [64], although not all studies can confirm this association [65]. The positive association of non-married couples sharing a common flat to T2DM can therefore be considered as very specific to the commuting belt around Berlin. Further research on an individual level is necessary to confirm this association.

Although several studies found an association between land-use, built environment and obesity and T2DM [66, 67], we found only a very moderate association between the proportion of built surfaces and T2DM. After carefully reviewing the results of a GWR model including the proportion of built surfaces as independent variable, we concluded that this association was misleading in our study area as it was only significant in the most sparsely populated area in Brandenburg. This seems implausible as villages in this area are generally very small and green spaces are widely available and accessible in walking distance. We thus excluded the proportion of built areas as independent variable from our analysis. However, this highlights the value of local regression models over global regression models to question the plausibility of possible associations.

We found no associations between availability of GPs and the prevalence of T2DM. Thus, access to and availability of GPs has no influence on the diagnosis of T2DM in our study area. Since the majority of T2DM is detected among persons in their 40 s and older [68], and diabetics in rural areas consulting GPs less frequently than diabetics in urban areas [69], it seems reasonable to assume that a substantial amount of diabetics in our study area only sought medical attention when symptoms of T2DM persisted as our population sample is older than the rest of northeastern Germany’s population. As a consequence, the number of undiagnosed diabetics in rural areas is potentially higher among middle-aged persons, which do not display any symptoms yet.

## Strengths and limitations

### Strengths

In this study, we used a large database, consisting of 1.8 million insurants. Our results clearly demonstrate that a spatial analysis using “big data” of health insurance providers is feasible and can be used to provide a finer spatial resolution for prevalence estimates of T2DM than it is currently possible with survey data.

Several spatial-epidemiological studies highlight the benefits of performing a cluster test based on point data over administrative data [30, 70, 71]. Detailed cluster detection based on point data could not only enhance prevention strategies [17, 30] but could also be used for a demand-driven allocation of healthcare facilities where they are needed most [48]. In northeastern Germany, this is of particular importance as the population is very unevenly distributed and the smallest administrative unit–municipalities–vary strongly in size and population among the states [72]. Further, Germany’s largest city Berlin counts as only one municipality. Five-digit postal codes were thus used for the sex- and age standardization to highlight intra-urban differences. German postal codes have the disadvantage of - specifically in predominantly rural regions - covering very large areas and are thus not very suitable for the allocation of future healthcare. As a consequence, our approach of combining a bivariate KDE with a cluster analysis may serve as an alternative and relative exact prioritization for allocating new GP resources in the near future.

### Limitations

First, our study was based on health insurance claims of northeastern Germany’s largest statutory health insurance provider. Although the AOK Nordost covers approximately one quarter of the population, the results cannot be assumed to sufficiently reflect the prevalence of T2DM for the whole population. Large socio-demographic differences exist between the insurants of the various statutory health insurance providers with the AOK having the largest proportion of persons with low income, low educational level and thus the highest prevalence of chronic diseases [25].

Second, we included all persons that were insured in 2012 with the AOK Nordost, irrespective of the length of insurance. We therefore did not exclude persons who died in 2012 from the analysis or persons being insured for short time-periods as these persons still contributed to the overall prevalence of T2DM.

Third, it is clear that the results of the bivariate KDE for T2DM represent the demographic distribution of insurants to a certain extent, given the strong association of T2DM to older age groups [3, 4, 22]. However, age-standardization is currently not available for a bivariate KDE in the CrimeStat IV software. As a consequence, the combined results of the bivariate KDE and the spatial scan statistic are more relevant for immediate allocation of GPs than for long-term planning of future healthcare.

Fourth, although most clusters were concentrated in areas with above-average prevalence estimates of the KDE, a small proportion of clusters was also concentrated in areas with below-average prevalence estimates. This is attributable to the different settings used in this study for the bivariate KDE and the spatial scan statistic. As we used an adaptive kernel for the KDE and a fixed radius of 10 km for the spatial scan statistic, higher prevalences cannot be sufficiently visualized if several hundred cases are concentrated in a very small location. This may occur for example with adjacent multi-story apartment blocks, which still constitute a significant cluster as detected by the spatial scan statistic but are smaller than the resolution offered by the KDE. When using fixed bandwidths of the same size for KDE and the spatial scan statistic simultaneously, this problem becomes less prominent [30].

Fifth, the associations examined in this study are based on aggregated data. Although our results generally reflect the results of other spatial-epidemiological studies on T2DM, it is necessary to review whether the associations revealed in this study at the ecological level are also valid associations on an individual level.

## Implications for future planning of healthcare

Our results clearly demonstrate that the prevalence of T2DM varies at very fine geographic scales. The small-scale spatial variability of T2DM thus challenges the applicability of the spatial scale of central areas (Mittelbereiche) at which the allocation of GPs is currently planned [7, 73]. Based on our results, a planning on smaller scales such as the association of municipalities would be more suitable to reflect the strong spatial variability of T2DM. It has been argued that the current provision of GPs–based on the ratio of 1 GP per 1671 inhabitants [7]—is too simplified and also outdated [8, 74]. The association of T2DM to location-specific socio-demographic population characteristics demands a strong deviation from these ratios and calls for a stronger acknowledgement of increased medical needs among the elderly and socially underprivileged populations. The revised planning guidelines of the federal association of statutory physicians in 2013 would allow deviations from the current ratio for areas with a particular high prevalence of diseases or specific socio-economic characteristics [75]. However, these revised planning guidelines still remain unspecific on how exactly a particular high prevalence or specific socio-economic characteristics can be translated into additional GP positions for a particular area. As a consequence, our analysis can only point out areas with a currently high medical demand and location-specific associations between T2DM and socio-demographic population characteristics.

Given that the spatial variability of T2DM is not only determined by current socio-demographic factors but also by the change of these factors over time [4], the results of our GWR analysis could serve as a first basis in developing approaches to model the expected, long-term future burden of T2DM to assist in allocating future GPs where they will be needed most.

## Conclusion

This is to date the largest small-scale spatial-epidemiological study of T2DM in northeastern Germany. Our results clearly show that T2DM varies at the very local level and that a large variation of T2DM prevalence can be explained by location-specific, socio-demographic population characteristics. Future planning of healthcare would greatly benefit from smaller spatial scales and need to deviate from simple inhabitants to GP ratios to reflect the increased prevalence of chronic diseases in older and socially underprivileged population groups. These results are therefore valuable for the future planning of healthcare in northeastern Germany. Our approach of analyzing the spatial distribution of chronic diseases at the very local level and geographically weighted regression is not only useful for northeastern Germany, but could be an effective way of targeting location-specific population groups with increased medical needs as precisely as possible in all countries, where chronic diseases are on the rise.

## Abbreviations

AIC: Akaike’s information criterion
AICc: Akaike’s corrected information criterion
BBSR: Federal Agency of Building and Urban Development
BIC: Bayesian information criterion
CV: cross validation
GIS: geographic information systems
GP: general practitioner
GWR: geographically weighted regression
ICD-10: international classification of disease, 10th revision
KDE: kernel density estimation
OLS: ordinary least squares
T2DM: type 2 diabetes mellitus
